# The Csr System Regulates *Escherichia coli* Fitness by Controlling Glycogen Accumulation and Energy Levels

**DOI:** 10.1128/mBio.01628-17

**Published:** 2017-10-31

**Authors:** Manon Morin, Delphine Ropers, Eugenio Cinquemani, Jean-Charles Portais, Brice Enjalbert, Muriel Cocaign-Bousquet

**Affiliations:** a LISBP, Université de Toulouse, CNRS, INRA, INSA, Toulouse, France; b Inria, University of Grenoble-Alpes, Grenoble, France; University of Florida; University of Washington

**Keywords:** Csr system, carbon metabolism, *Escherichia coli*, glycogen

## Abstract

In the bacterium *Escherichia coli*, the posttranscriptional regulatory system Csr was postulated to influence the transition from glycolysis to gluconeogenesis. Here, we explored the role of the Csr system in the glucose-acetate transition as a model of the glycolysis-to-gluconeogenesis switch. Mutations in the Csr system influence the reorganization of gene expression after glucose exhaustion and disturb the timing of acetate reconsumption after glucose exhaustion. Analysis of metabolite concentrations during the transition revealed that the Csr system has a major effect on the energy levels of the cells after glucose exhaustion. This influence was demonstrated to result directly from the effect of the Csr system on glycogen accumulation. Mutation in glycogen metabolism was also demonstrated to hinder metabolic adaptation after glucose exhaustion because of insufficient energy. This work explains how the Csr system influences *E. coli* fitness during the glycolysis-gluconeogenesis switch and demonstrates the role of glycogen in maintenance of the energy charge during metabolic adaptation.

## IMPORTANCE

Glycogen is a polysaccharide and the main storage form of glucose from bacteria such as *Escherichia coli* to yeasts and mammals. Although its function as a sugar reserve in mammals is well documented, the role of glycogen in bacteria is not as clear. By studying the role of posttranscriptional regulation during metabolic adaptation, for the first time, we demonstrate the role of sugar reserve played by glycogen in *E. coli*. Indeed, glycogen not only makes it possible to maintain sufficient energy during metabolic transitions but is also the key component in the capacity of cells to resume growth. Since the essential posttranscriptional regulatory system Csr is a major regulator of glycogen accumulation, this work also sheds light on the central role of posttranscriptional regulation in metabolic adaptation.

## INTRODUCTION

Metabolic adaptation describes the capacity to switch from one substrate to another. This mechanism is essential for the fitness and survival of microorganisms (1, 2). In the model bacterium *Escherichia coli*, metabolic adaptation influences the productivity of bioprocesses and also its persistence in the gut, with major consequences for human health. The persistence of commensal *E. coli* strains has already been shown to provide a barrier to infection in the intestine (3, 4). *E. coli* first consumes sugars and sugar derivatives through glycolytic pathways. In the absence of such substrates, it uses less favorable molecules such as acetate, succinate, or formate by activating the gluconeogenic pathway (1, 5). The mechanisms involved in pathogen exclusion in natural ecosystems are complex but notably involve competitive metabolic interactions with available nutriments (4, 6, 7). Since the availability of carbon substrates in the gut is constantly changing, metabolic adaptation and the capacity to switch from glycolysis to gluconeogenesis are essential for colonization and persistence of *E. coli* and hence for health (8).

Although the metabolic organization of *E. coli* is well described, metabolic adaptation is still not fully understood (9–11). In central carbon metabolism (CCM), the accumulation of several layers of regulatory mechanisms (transcriptional, posttranscriptional, translational, and posttranslational regulatory mechanisms) results in a complex and entangled control of carbon fluxes. At the posttranscriptional level (i.e., control of the stability and translation of mRNA), the main controller of CCM reported so far is the carbon storage regulator (Csr) system (12–16). This posttranscriptional controller is highly pleiotropic (17, 18) and is involved in the regulation of most adaptive phenomena, including virulence (19), motility (20, 21), stringent response (12), and glycogen synthesis (14, 22). Its main component is the conditionally essential protein CsrA, which is able to bind to target mRNAs, leading to either their degradation or their stabilization. CsrA activity is controlled through sequestration by CsrB and CsrC, two noncoding RNAs (ncRNAs) (23–25). Both of these ncRNAs are targeted by the protein CsrD, which triggers their RNase E-dependent degradation (26), through a molecular mechanism based on CsrD and CsrA antagonism (27). Transcription of CsrB and CsrC is activated by the BarA/UvrY two-component system (23, 25, 28) and is repressed by cAMP-CRP (29). The Csr system is said to positively regulate glycolysis while inhibiting gluconeogenesis (14–16, 30). It is hypothesized to be involved in the switch between the two metabolisms since the BarA/UvrY two-component system is needed for the efficient transition from glycolytic to gluconeogenic metabolism (2). The recently discovered circuitry linking catabolite repression and Csr regulatory systems (29) also suggests the involvement of CsrA in the transition, since the switch to gluconeogenic substrate consumption requires the alleviation of catabolite repression.

Here, we investigated the putative role of the Csr system in the metabolic adaptation from glycolysis to gluconeogenesis. Gene expression and metabolite pool analyses were performed with Csr system mutant strains throughout the glucose-acetate transition, which was considered a model of metabolic adaptation (9, 10, 31). We demonstrate the important role of the Csr system during this metabolic transition through its control of glycogen accumulation and cell energy homeostasis.

(Parts of this work were conducted as M.M.’s thesis project.)

## RESULTS

### The Csr system influences the dynamics of acetate reconsumption after glucose exhaustion.

**FIG 1:**
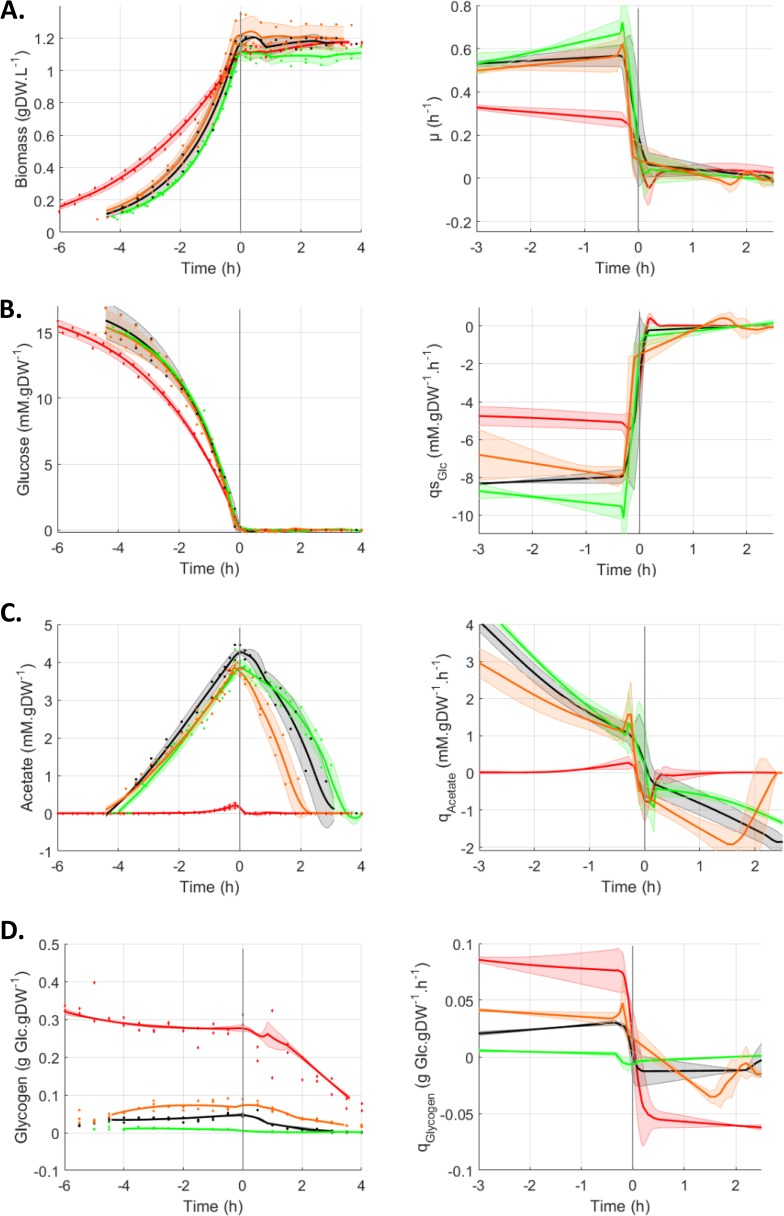
Behavior of Csr system mutants during the glucose-acetate transition. Cultures were monitored from 6 h before to 4 h after glucose exhaustion, which was set as time zero. All data concerning the replicates are displayed as dots, and the fitted average value of each strain is displayed as a line. Shaded areas represent ±1 standard deviation. WT, black circles; *csrBC* mutant, green squares; *csrD* mutant, orange triangles; *csrA51* mutant, red diamonds. (A) Changes in the biomass concentration (gDW ⋅ liter^−1^) and growth rate μ (h^−1^). (B) Extracellular glucose concentration (mM) and specific glucose production rates (mM ⋅ h^−1^ ⋅ gDW^−1^ ⋅ h^−1^). (C) Extracellular acetate concentration (mM) and specific acetate production rates (mM ⋅ h^−1^ ⋅ gDW^−1^ ⋅ h^−1^). (D) Glycogen concentration (ggluc ⋅ gDW^−1^) and specific glycogen production rates (ggluc ⋅ h^−1^ ⋅ gDW^−1^ ⋅ h^−1^).

The glucose-acetate transition is a model of adaptation from glycolytic to gluconeogenic metabolism (9, 10, 31). Briefly, this transition consists of *E. coli* cells growing exponentially on a glucose minimal medium and excreting acetate into the medium as a metabolic overflow product. When the cells run out of glucose, the CCM is reorganized, thereby enabling the cell to consume the acetate. The behaviors of three strains with modified CsrA activities (*csrA51*, Δ*csrBC*, and Δ*csrD* mutant strains) were investigated. Deletions of CsrB and, to a lesser extent, of CsrC ncRNAs are known to increase CsrA activity by preventing its sequestration (24, 25). Deletion of *csrD* triggers an increase in CsrB and CsrC ncRNA concentrations, which promotes the sequestration of CsrA and reduces its activity (26). In the *csrA51* mutant strain, the deletion of the last 11 amino acids of CsrA has been reported to dramatically reduce its activity while maintaining strain viability. This construction has been widely used as the gold standard to explore the function of CsrA (17, 32, 33). To investigate the glucose-acetate transition, the three mutants and their wild-type (WT) isogenic control were grown dynamically in controlled bioreactors in M9 minimal medium supplemented with 0.27% glucose (9). The biomass and extracellular glucose and acetate concentrations were measured at 30-min intervals (Fig. 1). The growth of all four strains was exponential during glucose consumption (Fig. 1A). As expected from previous work (16), the *csrA51* mutant’s growth rate on glucose was lower than that of the WT (Fig. 1A). No significant differences were observed between the WT and the Δ*csrBC* and Δ*csrD* mutant strains during the growth phase on glucose (Fig. 1A). This was corroborated by the similar glucose consumption profiles of the strains (Fig. 1B). The *csrA51* mutant strain produced a very low level of acetate during growth on glucose in comparison with the three other strains, whose production rates and final levels of acetate were similar (Fig. 1C). The small amount of acetate in the *csrA51* mutant strain was completely consumed in the minutes following glucose exhaustion. The other three strains also consumed the acetate produced after glucose exhaustion but with different timings, resulting in the Δ*csrD* mutant strain consuming its acetate first, followed by the WT and then the Δ*csrBC* mutant strain (Fig. 1C). Accordingly, during the acetate consumption phase, higher rates of acetate consumption were observed in the Δ*csrD* mutant strain, followed by the WT and then the Δ*csrBC* mutant strain (Fig. 1D). To illustrate, at 1 h after glucose exhaustion, these rates were, respectively, −1.50 ± 0.26, −0.82 ± 0.32, and −0.58 ± 0.12 mM ⋅ g of dry weight (gDW)^−1^ ⋅ h^−1^ in the Δ*csrD* mutant, WT, and Δ*csrBC* mutant strains. We detected no difference in biomass content that could be related to the differences in the timing of acetate consumption (Fig. 1A).

Since the *csrA51* mutant strain is reported to overaccumulate glycogen (14, 16, 22), we assessed the concentration of this storage polysaccharide in the cell during the glucose-acetate transition (Fig. 1D). As expected (22), the *csrA51* mutant strain accumulated up to 0.28 g of glucose (ggluc) ⋅ gDW of glycogen^−1^, representing about 30% of the biomass dry weight. Glycogen also accumulated in the Δ*csrD* mutant cells but to a lesser extent (maximum of 0.07 ggluc ⋅ gDW^−1^, representing 7% of the cell dry weight) and even less in the WT (4% of the cell dry weight). In contrast, the Δ*csrBC* mutant cells stored a very small quantity of glycogen (maximum of 0.01 ggluc ⋅ gDW^−1^, representing about 1% of the biomass). The glycogen content in the four strains remained mostly stable during the exponential phase of growth on glucose (concentrations and rates remained constant), and all four strains used glycogen after glucose exhaustion. Overall, glycogen production and consumption rates were high in the *csrA51* mutant strain (respectively, 0.078 ± 0.014 and −0.057 ± 0.014 gglc ⋅ gDW^−1^ ⋅ h^−1^ 1 h before and 1 h after glucose exhaustion), medium in the Δ*csrD* mutant (0.035 ± 0.004 and −0.018 ± 0.001 gglc ⋅ gDW^−1^ ⋅ h^−1^), and low in the Δ*csrBC* mutant (0.003 ± 0.001 and −0.002 ± 0.001 gglc ⋅ gDW^−1^ ⋅ h^−1^) compared to those of the WT (0.028 ± 0.001 and −0.012 ± 0.007 gglc ⋅ gDW^−1^ ⋅ h^−1^).

To conclude, the investigated strains showed a range of phenotypes according to their CsrA activity. The *csrA51* mutant was the most affected strain, with high glycogen and low acetate contents and a reduced growth rate. The Δ*csrD* mutant also had a higher glycogen content than the WT and accelerated acetate consumption. The Δ*csrBC* mutant displayed the reverse phenotypes (low to no glycogen content and delayed acetate consumption). In addition to its effect on growth rates and glycogen accumulation, the Csr system is involved in the dynamics of the glycolysis-gluconeogenesis transition, notably through its impact on acetate consumption after glucose exhaustion.

### CCM gene expression is differently influenced by the Csr system before and after glucose exhaustion.

**FIG 2:**
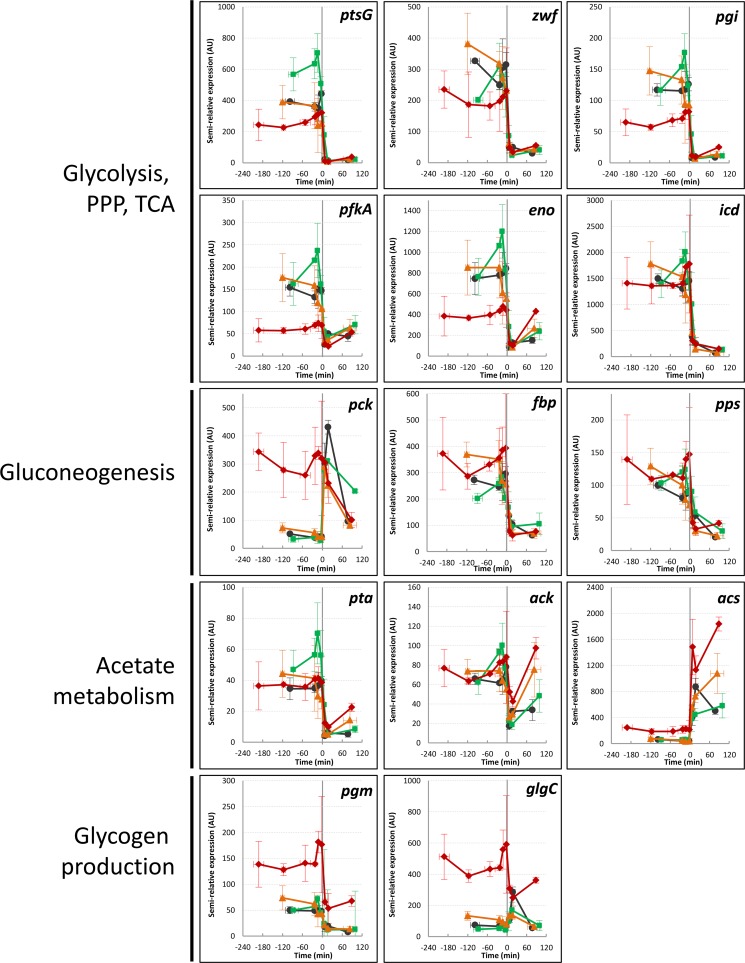
Expression of key metabolic genes during the glucose-acetate transition of Csr system mutants. Gene expression (relative to the *idnT* RNA reference) was investigated from 3 h before to 2 h after glucose exhaustion. WT, black circles; *csrBC* mutant, green squares; *csrD* mutant, orange triangles; *csrA51* mutant, red diamonds. *ptsG*, glucose phosphotransferase system permease PtsG subunit (European Bioinformatics Institute European Nucleotide Archive accession no. EG10787); *pgm*, phosphoglucomutase (accession no. EG12144); *glgC*, glucose-1-phosphate adenylyltransferase (accession no. EG10379); *zwf*, glucose 6-phosphate-1-dehydrogenase (accession no. EG11221); *pgi*, phosphoglucose isomerase (accession no. EG10702); *pfkA*, phosphofructokinase (accession no. EG10699); *eno*, enolase (accession no. EG10258); *icd*, isocitrate dehydrogenase (accession no. EG10489); *pck*, phosphoenolpyruvate carboxykinase (accession no. EG10688); *pta*, phosphate acetyltransferase (accession no. EG20173); *ack*, acetate kinase (accession no. EG10027); *ACS*, acetyl coenzyme A synthetase (accession no. EG11448); *pck*, phosphoenolpyruvate carboxykinase (accession no. EG10688); *fbp*, fructose 1,6-bisphosphatase (accession no. EG10283). The values are averages of three replicates with error bars representing standard deviations. PPP, pentose phosphate pathway; TCA, tricarboxylic acid cycle.

We assumed that the differences in acetate consumption between the strains could result from differential gene expression in the CCM after glucose exhaustion. This was assessed by performing quantitative reverse transcription (RT)-PCR analysis of 14 key CCM genes (34) during the metabolic transition from 3 h preceding to 2 h after glucose exhaustion (Fig. 2). During the glucose consumption phase, massive differences in gene expression were observed between the mutant strains and the WT, especially the *csrA51* mutant strain (as previously described by Morin et al. [16]). In contrast, the differences between the strains were globally reduced after glucose exhaustion, except for the genes involved in acetate and glycogen metabolism (Fig. 2). For acetate metabolism (*ACS*, *ack*, and *pta*), higher expression was noticed in the *csrA51* and Δ*csrD* mutant strains than in the WT, which could be consistent with their improved acetate consumption rates. The expression of glycogen-related genes (*pgm* and *glgC*) was increased, but only in the *csrA51* mutant strain. We thus conclude that the Csr system controls different sets of genes before and after glucose exhaustion, the later set being devoted to the use of the remaining carbon sources (acetate and glycogen).

### Analysis of the metabolite pools revealed different energy status in the Csr system mutant strains after glucose exhaustion.

**FIG 3:**
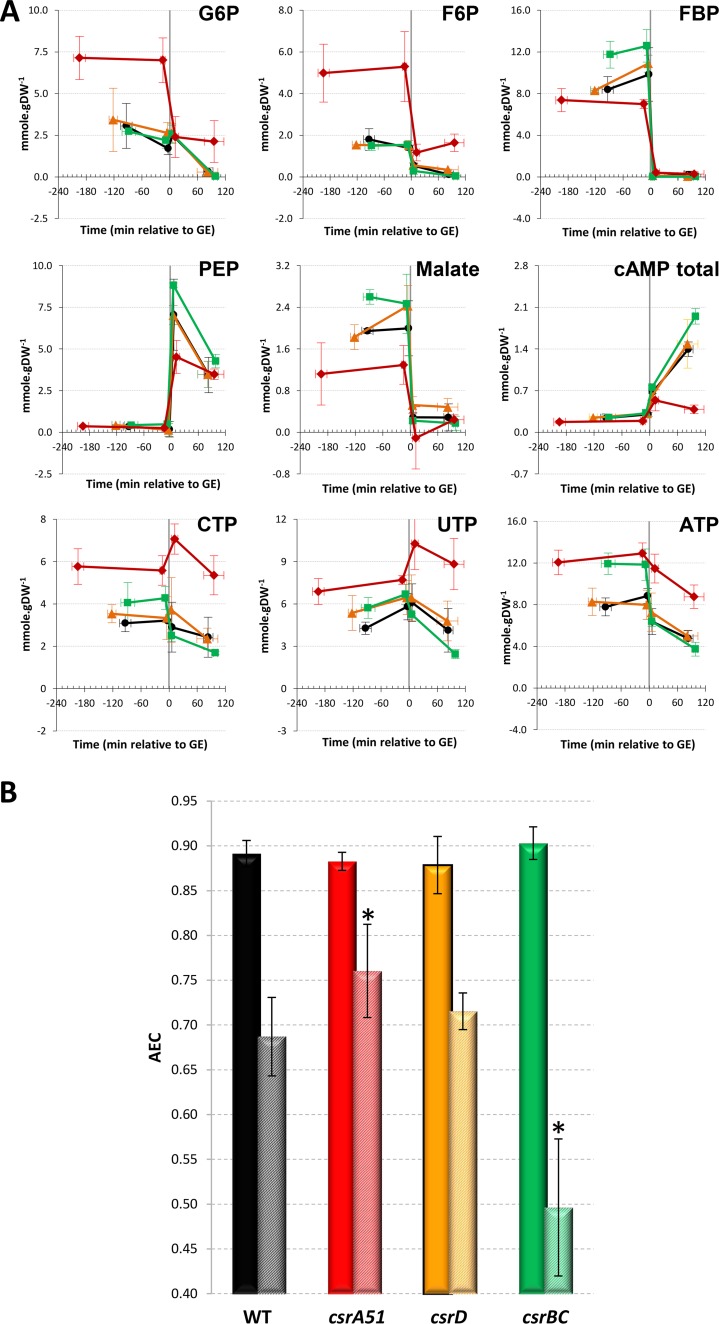
Concentrations of key central carbon metabolites during the glucose-acetate transition of Csr system mutants. (A) Metabolite concentrations were investigated from 3 h before to 2 h after glucose exhaustion. WT, black circles; *csrBC* mutant, green squares; *csrD* mutant, orange triangles; *csrA51* mutant, red diamonds. PEP, phosphoenolpyruvate. (B) The AECs of the same four strains during glucose consumption (plain bars) and 1.5 h after glucose exhaustion (striped bars) are shown. A significant difference between a mutant and the WT is represented by an asterisk (*P* < 0.05 [*t* test]). The values in both panels are averages of three independent biological replicates with error bars representing standard deviations.

We then measured key metabolite pools in the CCM over time in the four strains by using liquid chromatography-tandem mass spectrometry (LC-MS/MS) (Fig. 3). The profiles of the metabolites assessed over time were mostly similar in the four strains, with a drop in concentrations after glucose exhaustion, except for the alarmone cAMP and for phosphoenolpyruvate, as previously described in the WT strain (9). Before glucose exhaustion, the Δ*csrBC* mutant strain had higher concentrations of fructose 1,6-bisphosphate (FBP) and malate, whereas the Δ*csrD* pools were mostly comparable to those of the WT (Fig. 3A). As expected (16), the *csrA51* mutant strain displayed larger metabolite pools in the upper part of the glycolysis process (glucose-6-phosphate [G6P], fructose-6-phosphate [F6P]) than the WT. These pools decreased after glucose exhaustion but, unexpectedly, were maintained in the *csrA51* mutant strain at levels equivalent to those measured in the WT strain during the glucose consumption phase (Fig. 3A). Likewise, the nucleotide triphosphate pools remained high in the *csrA51* mutant strain even after glucose exhaustion.

These results suggested that the energy level after glucose exhaustion differs between strains. We therefore investigated the energy status of the cells by calculating the adenylate energy charge (AEC) as an index of the bacterium metabolic state (35). The AEC on glucose was between 0.87 and 0.91 in all of the strains during the glucose consumption phase (Fig. 3B). Such values are expected in exponentially growing cells (35). At 90 min after glucose exhaustion, the AEC had decreased in all of the strains. However, in the *csrA51* mutant strain, the AEC remained significantly higher than in the WT, and the Δ*csrBC* mutant AEC collapsed to 0.50, a value previously linked to dying cells (35). In conclusion, the *csrA51* mutant strain maintained high energy levels after glucose exhaustion, whereas there was a dramatic drop in the energy status of the Δ*csrBC* mutant strain. We thus hypothesize that the energy levels in the Csr system mutant strains could be linked to the observed differences in the kinetics of acetate consumption.

### Both glycogen and acetate influence the energy status after glucose exhaustion.

**FIG 4:**
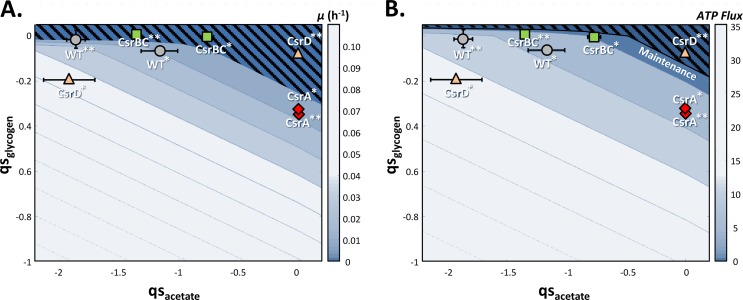
Metabolic capacities of the WT and Csr system mutant strains during growth on glycogen and acetate. Flux balance analysis of the four strains was performed with glycogen and acetate as carbon sources by using two different objective functions, biomass and ATP production (see Materials and Methods for details). (A) Maximum predicted growth rates (μ) for various pairs of glycogen and acetate uptake rates (qs). (B) Maximum flux of ATP predicted by the model. Bars to the right of each plot indicate the color code used for the growth rate (A) and ATP flux (B) values. The four strains, the WT (gray circles), the *csrBC* mutant (green squares), the *csrD* mutant (orange triangles), and the *csrA51* mutant (red diamonds), were assessed at two different times 90 min (*) and 150 min (**). The values are averages of three replicates with error bars representing standard deviations.

We analyzed the relationship between energy fluxes and acetate consumption rates by investigating the distribution of the energy fluxes after glucose exhaustion in the different strains. During the acetate consumption phase, glycogen is also consumed as an intracellular storage sugar and is thus also likely to be involved in the balance between energy fluxes. To examine the influence of glycogen and acetate flux on the growth rate and ATP flux, we used a previously developed constraint-based model of *E. coli* (16, 36). These parameters were analyzed in the ranges of acetate and glycogen consumption rates we monitored after glucose exhaustion (Fig. 1; see Materials and Methods for details of the procedure). We compared the ATP fluxes predicted by the model with cell maintenance that we estimated at 2.9 mmol ⋅ gDW^−1^ ⋅ h^−1^ from experimental data obtained with the WT strain (32) and considered to be the same in all four strains. The results of analysis of the flux balance are shown in Fig. 4. Acetate and glycogen uptake rates were insufficient to detect growth (µ < 0.05 h^−1^) in any of the four strains (Fig. 4A), since ATP production barely covered the maintenance costs (Fig. 4B). At 2.5 h after glucose exhaustion, the WT and Δ*csrBC* mutant strains produced ATP from acetate in the absence of glycogen (the ATP fluxes produced ranged between 5 and 12 mmol ⋅ gDW^−1^ ⋅ h^−1^), while in the *csrA51* mutant strain, ATP was produced from glycogen in the absence of acetate. Although the Δ*csrD* mutant strain displayed the greatest ATP flux from a mixture of glycogen and acetate 90 min after glucose exhaustion (Fig. 4A), growth was only barely detectable and stopped 1 h later when both acetate and glycogen were depleted (Fig. 1 and 4). We thus conclude that both acetate and glycogen can be used to maintain the energy status of the cells after glucose exhaustion but that their consumption rates are too low to ensure observable growth. Even in the *csrA51* mutant strain, which has the highest energy level AEC and glycogen content (up to 30% of the biomass), the ATP produced is only sufficient to ensure maintenance, i.e., to allow the strain to survive but not to grow efficiently.

### Suppression of glycogen synthesis hinders the capacity of the *csrA51* mutant strain to maintain high energy levels after glucose exhaustion.

**FIG 5:**
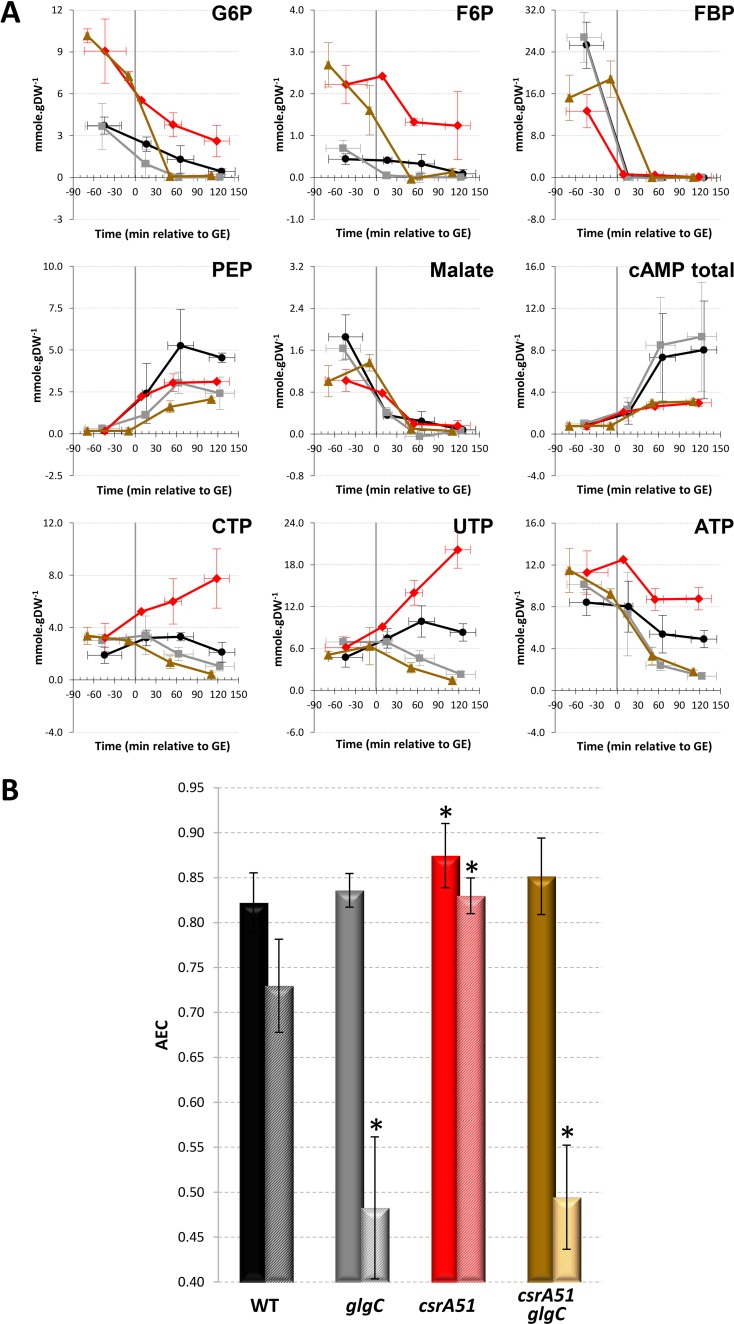
Concentrations of key central carbon metabolites throughout the glucose-acetate transition of Csr system and/or glycogen-deficient mutants. (A) Metabolite concentrations were investigated from 3 h before to 2 h after glucose exhaustion. WT, black circles; Δ*glgC* mutant, gray squares; *csrA51* mutant, red diamonds; *csrA51* Δ*glgC* mutant, brown triangles. PEP, phosphoenolpyruvate. (B) The AECs of the same four strains during glucose consumption (plain bars) and 90 min after glucose exhaustion (striped bars) are shown. A significant difference between a mutant and the WT is represented by an asterisk (*P* < 0.05 [*t* test]). The values are averages of three independent biological replicates with error bars representing standard deviations.

As shown above, both acetate and glycogen can be used to maintain the energy status after glucose exhaustion but the corresponding ATP fluxes did not enable us to distinguish differences in the energy level AEC and fitness related to acetate consumption in the different strains. Only glycogen levels appeared to accompany the high energy level of the *csrA51* mutant strain and the low energy level of the Δ*csrBC* mutant (Fig. 1D and 3B). We therefore hypothesized that the impact of Csr on the energy status can only be mediated by the impact of CsrA on glycogen accumulation. To confirm this hypothesis, we prevented glycogen synthesis in the WT and *csrA51* mutant strains by deleting the *glgC* gene. The same pools of metabolites as those shown in Fig. 3 were then assessed during the glucose-acetate transition in the WT and Δ*glgC*, *csrA51*, and *csrA51* Δ*glgC* mutant strains (Fig. 5A). Some of the WT and *csrA51* mutant metabolite pools, e.g., FBP, differed quantitatively from those shown in Fig. 3A, very likely because these cultures were performed in Erlenmeyer flasks instead of in reactors with regulated pH and monitored oxygenation. However, the trends were similar under both conditions. Remarkably, the deletion of *glgC* prevented the maintenance of elevated G6P, F6P, and nucleotide triphosphate in the *csrA51* mutant strain. Prevention of glycogen synthesis did not affect the AEC before glucose exhaustion (Fig. 5B). After glucose exhaustion, the AEC of the two strains unable to store glycogen dropped drastically in comparison to that of the WT and *csrA51* mutant strains. We therefore conclude that the maintenance of the energy status after glucose exhaustion in the *csrA51* mutant strain results from its high glycogen content.

### The Csr system affects cellular fitness through its control of glycogen synthesis.

**FIG 6:**
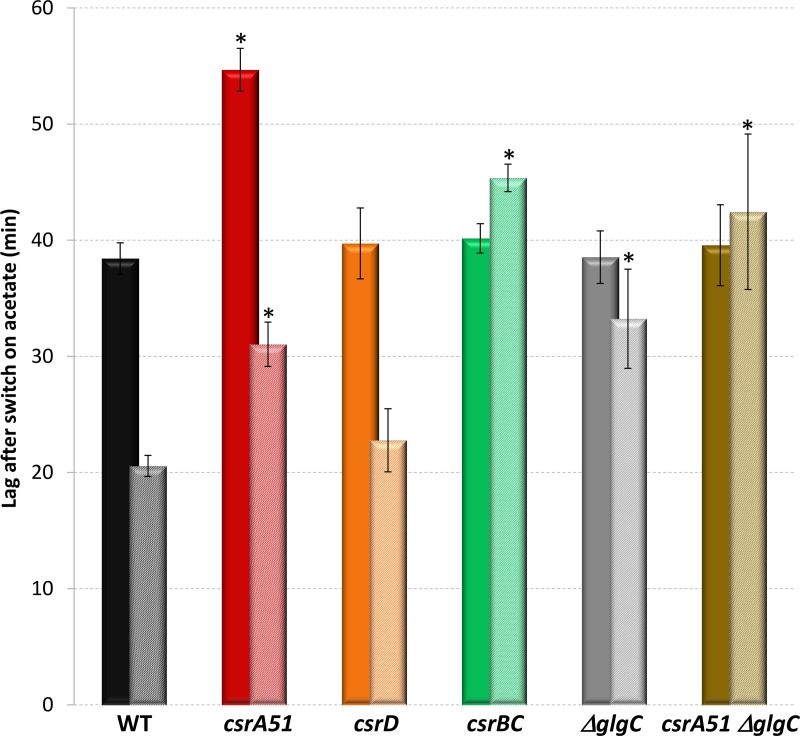
Delay before the full growth rate was reached on acetate. For each strain, cells from a mother culture on M9-glucose were transferred to M9-acetate and the time lag until full growth on the new substrate was measured. Cells were sampled either 60 min before (plain bars) or 60 min after (striped bars) glucose exhaustion. The data are averages of three or more independent biological replicates with error bars representing standard deviations. A significant difference between a mutant and the WT is represented by an asterisk (*P* < 0.05 [*t* test]).

Taken together, our results suggest that the Csr system influences the energy status through the control of glycogen synthesis. This affects the ability of the strain to switch to acetate metabolism after glucose exhaustion. To demonstrate this scenario, we investigated the dynamics of the metabolic adaptation of the cells when switched to fresh medium containing acetate as the sole carbon source. The capacity of Csr and/or glycogen mutant cells to resume growth on fresh acetate minimum medium was assessed before and after glucose exhaustion. The times required to recover full growth on acetate were determined (Fig. 6). As previously demonstrated (31), the WT strain required 38 min to switch from glucose- to acetate-based growth. This delay was reduced to 21 min when the cells were sampled 1 h after glucose exhaustion, likely because of the induced expression of gluconeogenic enzymes. The delays were similar in the Δ*csrD* mutant strain. In the *csrA51* mutant strain, the delays increased by 40 to 50% but the same relative decrease was observed after glucose exhaustion. The long delays in the *csrA51* mutant strain could be explained by the difficulty in setting up acetate consumption in the presence of highly abundant glycogen, which could be a more favorable substrate. The delay in the Δ*csrBC* mutant strain growing on glucose was similar to that in the WT strain. In contrast, the delay in the Δ*csrBC* mutant strain was not reduced after glucose exhaustion. This property was also observed in the Δ*glgC* and *csrA51* Δ*glgC* mutant strains. The increase in the delays in the *csrA51* mutant strain switched to acetate was also suppressed in the *csrA51* Δ*glgC* mutant strain. Since the glycogen level in the *csrBC* mutant strain was zero after glucose exhaustion (Fig. 1D), we conclude that glycogen is necessary to start acetate uptake 1 h after glucose exhaustion. Therefore, the Csr system affects the establishment of metabolic adaptation after glucose exhaustion through its control of glycogen synthesis.

## DISCUSSION

The Csr system was recently demonstrated to be a major controller of upper glycolysis fluxes (16), but its involvement in metabolic adaptation is less clear in the literature. CsrA is known to positively regulate glycolytic genes and negatively regulate gluconeogenic genes (15, 16). A study of the BarA/UvrY two-component system during the metabolic switch suggested that the Csr system is crucial for efficient adaptation between different metabolic pathways (2). Here, we showed that gene expression in the CCM (glycolysis, gluconeogenesis, the pentose phosphate pathway, and the tricarboxylic acid cycle) did not present strong discrepancies between the Csr system mutants during the acetate consumption phase, in deep contrast to the situation during glucose consumption. It will be awkward to totally rule out any control of these genes by CsrA, since regulation could be at the posttranscriptional level. The control by CsrA could also be counterbalanced by its higher sequestration by CsrB, since the latter level has been reported to increase during the transition (23, 37). Most importantly, CsrA was demonstrated to control the expression of genes encoding functions in acetate and glycogen metabolism after glucose exhaustion. This gene expression profiling, combined with acetate measurements and switch experiments, demonstrated that CsrA influences the timing of acetate and glycogen utilization after glucose exhaustion. We proved that glycogen allows the maintenance of a minimal cellular energy level after glucose exhaustion. The energy related to glycogen and/or acetate catabolism provided the energy required for maintenance but was not sufficient to sustain growth on glycogen alone. We also showed that glycogen accumulation increased cell fitness by allowing a rapid restart on acetate. In the *csrA51* mutant strain, the overaccumulation of glycogen was also accompanied by levels of G6P and F6P and energy status after glucose exhaustion matching those of the WT during growth on glucose. Conversely, a Δ*csrBC* mutant strain that accumulates a very low level of glycogen presents phenotypes similar to those of a Δ*glgC* mutant strain after glucose exhaustion, with a low energy status and a weaker adaptive capacity to use acetate. From our work, it is now clear that CsrA regulates the metabolic transition from glycolysis to gluconeogenesis in *E. coli*.

Most of the elements presented here strengthen the hypothesis that the transition control by the Csr system is exerted through the regulation of glycogen synthesis and its utilization. The only clue we observed concerning a glycogen-independent effect of CsrA during the transition is in the production of cAMP, an alarmone that has an important function in the catabolite repression process (38). Indeed, a *csrA51* mutant strain induced cAMP production after glucose exhaustion, but the levels were about five times lower than in the WT (Fig. 5A). This could result from the greater availability of G6P, since this substrate indirectly controls adenylate cyclase activity (39). However, the *csrA51* Δ*glgC* mutant strain presents the same phenotype, proving that this lower cAMP level does not result from the greater glycogen content and is therefore directly linked to CsrA activity. CsrA could thus affect important metabolic adaption processes, such as catabolite repression, independently of glycogen content. This reinforces the very recently discovered link between Csr and catabolite repression regulatory systems (29). This new regulation was shown to be related to the control of the expression of the CsrB and CsrC ncRNAs via the main catabolic repression actors EIIA^Gluc^ and cAMP-CRP. There could be another clue to glycogen-independent control; i.e., if glycogen exhaustion hinders metabolic adaptation, glycogen overaccumulation (as in the *csrA51* mutant strain) should favor it. However, the delay before the beginning of acetate anabolism was longer in the *csrA51* mutant strain. This phenotype is very likely explained by the fact that glycolysis is still supplied in G6P by glycogen after glucose exhaustion in the *csrA51* mutant and this could impair/prevent the establishment of gluconeogenic metabolism. Therefore, here again, this phenotype is linked to glycogen accumulation. We consequently conclude that CsrA control of metabolic adaptation is mediated mainly through glycogen accumulation.

Given its structural characteristics and its metabolic regulation, glycogen was hypothesized to be a storage compound providing both carbon and energy for microorganisms (40). However, no demonstration of this function was previously reported in the literature (41–43). Here, using a metabolomics approach, we proved for the first time that glycogen is essential to maintain the cell energy level after glucose exhaustion. This is especially important since it was postulated that to classify glycogen as an energy storage compound, its use as a supply for energy maintenance had to be demonstrated (40). With this work, this is now the case. Glycogen metabolism was also linked to environmental survival (43). It was recently hypothesized and backed up by modeling that glycogen is an energy supply for immediate use in the following growth phase of *E. coli* (42). Here again, we experimentally demonstrated the validity of these *in silico*-based conclusions by showing that metabolic adaptation of cells depends on glycogen content. Thus, this work also provides significant clues to the function of glycogen in *E. coli*.

To conclude, this work is of particular importance in understanding (i) the function of CsrA-mediated posttranscriptional control, (ii) the role of glycogen in bacteria, and (iii) the mechanisms behind metabolic adaptation and fitness. This knowledge is crucial not only for control of the ability of *E. coli* to efficiently colonize the gut or other microbial communities and environments but also for the optimization of biotechnological processes in which the metabolic flexibility of *E. coli* is extensively exploited for the use of a broad range of substrates.

## MATERIALS AND METHODS

### Strains.

All of the strains used in this study came from *E. coli* K-12 MG1655 (WT). The *csrA51* mutant strain was built by deleting the last 11 amino acids of the CsrA protein by λ Red system recombination (17, 32). The *csrD* mutant strain was constructed by completely deleting the *csrD* gene by λ Red system recombination (17). Likewise, the *csrBC* mutant strain was constructed by completely deleting the CsrB and CsrC ncRNAs by λ Red system recombination. The Δ*glgC* and *csrA51* Δ*glgC* mutants were obtained from the JW3393 strain (Keio collection [44]) by moving the mutation (Δ*glgC*::*Kan*) to the WT or the background of the *csrA51* mutant strain by bacteriophage P1-mediated transduction.

### Growth conditions.

Bioreactor cultures were performed with a volume of 500 ml of M9 medium complemented with 2.7 g ⋅ liter^−1^ glucose with pH and oxygenation controls (16). Three biological replicates were produced per strain (two for the *csrA51* mutant strain). The optical density at 600 nm (OD_600_) of extra- and intracellular samples was measured at 30-min intervals throughout the culture period. Flask cultures were performed with 200-ml baffled Erlenmeyer flasks with a total volume of 50 ml of M9 medium supplemented with 2.7 g ⋅ liter^−1^ glucose. All of the cultures were performed at 37°C.

### Extracellular and intracellular metabolites.

The extracellular metabolites were identified and quantified by H^+^ high-performance liquid chromatography (HPLC) (Agilent Technologies 1200 Series HPLC and an Aminex HPX-87h column to separate acid and sugar). The analysis was carried out at 48°C with 5 mM H_2_SO_4_ as the eluent. Intracellular metabolites were sampled at four different times during culture by the differential method (45). The metabolic intracellular content was assessed by LC-MS/MS as previously described (16). The AEC was quantified as previously described (35). Specific growth rates and consumption and production rates were determined as previously described (46).

### RT-PCR analysis.

Gene expression levels were analyzed by quantitative PCR (EvaGreen; Biotium, CA) on a Biomark 96.96 Dynamic Array (Fluidigm, CA) as previously described (16). Briefly, the equivalent of 5 mg of cell dry weight was harvested and directly flash frozen in liquid nitrogen at the same time as the intracellular metabolites. RNA extraction and RT were performed as previously described (32). The transcripts levels were normalized to the *idnT* reference gene used as a constitutive reference (47).

### Quantification of intracellular glycogen.

Intracellular glycogen was quantified as previously described (48). Briefly, cells were lysed and glycogen was hydrolyzed into glucose subunits by an amyloglucosidase. The glucose subunits were then quantified with the Glucose (GO) Assay kit (Sigma-Aldrich, France).

### Flux balance analysis.

The genome scale model and constraints used in this study were previously described (16), except for the lower boundary of the non-growth-associated maintenance flux, which was set to 2.9 mmol ⋅ gDW^−1^ ⋅ h^−1^. The model is a slightly modified version of the genome scale reconstruction iAF1260-flux2 (36). Flux balance analyses were performed with the COBRAv2 toolbox with GLPK as the linear programming solver (49). The glucose uptake rate was set to zero, while the oxygen uptake rate was set to a limiting value of −15 mmol ⋅ gDW^−1^ ⋅ h^−1^ to allow growth on acetate. The acetate and glycogen uptake rates were varied within the range of values monitored after glucose exhaustion (see Fig. 1), and for each pair of uptake rates, either the biomass or the ATP flux was optimized (with the lower boundary of the maintenance flux set to zero in the latter case).

### Switch experiments.

To investigate the timing of phenotypic adaptation, cell samples were collected from a “mother” culture 60 min before and 60 min after glucose exhaustion as previously described (31). Briefly, cell samples were rapidly filtered, rinsed, and used to inoculate 250-ml Erlenmeyer flasks filled with 30 ml of acetate medium. Growth of the “daughter” cultures was monitored by measuring the OD_600_ by spectrophotometry at 0, 15, 80, and 120 min after inoculation. We made sure that the cells from the daughter cultures reached the maximal growth rate (μ_max_) by measuring the growth rate between 80 and 120 min. The lag time was calculated as previously described (31).
